# Cystoid Macular Edema Related to Uncomplicated Cataract Surgery and Topical Prostaglandin Analogs: A Systematic Review of Randomized Controlled Trials

**DOI:** 10.7759/cureus.72920

**Published:** 2024-11-03

**Authors:** Ana Betzaida Santamaria, Lautaro Vera, Fernanda Rebollo, Curt Hartleben-Matkin

**Affiliations:** 1 Ophthalmology Glaucoma, Fundación de Asistencia Privada Conde de Valenciana, IAP, Ciudad de Mexico, MEX; 2 Ophthalmology Glaucoma, Panama Eye Center, Ciudad de Panama, PAN

**Keywords:** anti-glaucoma medications, glaucoma therapy, phacoemulsification cataract surgery, prostaglandin analogue, pseudophakic cystoid macular edema

## Abstract

Introduction: Cystoid macular edema (CME) presents with thickening of the macula with an accumulation of fluid due to breakage of the inner and outer blood-retinal barriers. It is referred to as pseudophakic CME (PCME) if it occurs after cataract surgery. PGF2a is a prostaglandin analog (PGA) used to lower intraocular pressure (IOP). In uncomplicated cataract surgery, the cumulative effect of topical PGA can make clinically significant PCME more prevalent. There are conflicting data regarding the association of PCME and the use of PGA in the perioperative period of uncomplicated cataract surgery, and its use remains controversial. We aim to determine the effect of PGA usage in the perioperative period in cases of uncomplicated cataract surgery on the development of PCME.

Methodology: A systematic review following the Preferred Reporting Items for Systematic Reviews and Meta-Analyses (PRISMA) in seven electronic databases was performed. The main outcome measure included the development of PCME diagnosed by clinical examination, OCT, or FA of adult patients (age > 16 years) undergoing routine cataract surgery using PGA in the perioperative period reported in randomized controlled trials.

Results: The search returned 196 articles, and 155 unique citations were identified after removing duplicates. According to the inclusion and exclusion criteria, four studies were selected for our review. They did not find a measurable effect on PCME incidence by PGA in the perioperative period in patients undergoing cataract surgery.

Conclusion: The results show no causal relationship between PCME in patients using PGA undergoing uneventful cataract surgery, suggesting they do not have to be suspended in patients without known risk factors of PCME.

## Introduction and background

Introduction

Cystoid macular edema (CME) is a condition characterized by the thickening of the macula secondary to the accumulation of fluid due to the breakage of the inner and outer blood-retinal barrier causing an increase in perifoveal capillary permeability by inflammatory mediators [1]. The pathology picture of CME is the presence of cystic changes in the outer plexiform layer (OPL) causing the classic “petaloid” leakage secondary to the structure of the Henle fibers. Still, structural changes studied with optical coherence tomography (OCT) show that the changes start in the inner nuclear layer (INL). They progress to involve the OPL and eventually develop subretinal fluid [1].

Cataracts are the leading cause of reversible blindness worldwide [2]; therefore, cataract extraction is one of the most prevalent surgeries performed worldwide [3]. When CME occurs after cataract surgery, it is referred to as pseudophakic CME (PCME) or Irvine-Gass syndrome, causing a decreased visual acuity in the early postoperative period, the peak incidence observed at the five-week postoperative period [4].

PCME can be diagnosed clinically when a patient complains of decreased visual acuity in the postoperative period of cataract surgery, and an OCT of the macula reveals intraretinal accumulation of fluid as cystoid hyporeflective cavities with an increase in the central macular thickness (CMT) [5]. The incidence of clinically significant PCME is 1%-2% [6,7]. On the other hand, the incidence of asymptomatic PCME which is not clinically significant and is only diagnosed by imaging modalities is higher, such as fluorescein angiography (FA) (16% of cases) [8] and OCT (41%of cases) [9].

Among all the available imaging techniques, OCT is the gold standard in the diagnosis of PCME since it provides quantitative data to evaluate the presence of edema by measuring the CMT and has replaced FA by proving to have a higher sensitivity to detect macular edema, in addition to its noninvasive methodology [10].

Prostaglandins are lipids produced from the breakup of phospholipids by phospholipase A2 resulting in arachidonic acid. Arachidonic acid is then metabolized to thromboxanes and prostaglandins by the cyclooxygenase enzymes [11]. PGF2a is a prostaglandin analog (PGA) commonly used in ophthalmology to lower the intraocular pressure (IOP) in patients with ocular hypertension (OH) or glaucoma by increasing the uveoscleral outflow of the aqueous humor through the extracellular matrix remodeling of the trabecular meshwork [12]. The commercially available PGA are latanoprost, bimatoprost, travoprost, and tafluprost; they are first-line medical treatment options for patients with glaucoma and OH [13].

Risk factors associated with PCME identified in a large retrospective database study are diabetes, capsular rupture with or without vitreous loss, a previous diagnosis of epiretinal membrane, uveitis, retinal vein occlusion, retinal detachment repair, older age, and male sex [6,7].

There is controversial data regarding the association of PCME and the use of PGA in the perioperative period of uncomplicated cataract surgery. Some studies report a greater risk of PCME after uncomplicated cataract surgery [6,14-16], and others did not show an increased risk [17-20]. This is reflected by the absence of official guidelines that would assist surgeons in the perioperative period of PGA users, and a great number of surgeons (65%) report discontinuing PGA perioperatively, regardless of the circumstances [21]. A survey of ophthalmologists from the UK reported that 60% continued using PGA after cataract surgery, 20% reported they did not use them routinely, and 20% reported that they suspended it if there were risk factors for PCME [17]. This proves that a gap in knowledge regarding this is a common issue, and the use of PGA perioperatively in cataract surgery remains controversial.

## Review

Aims and hypothesis

We aimed to determine the effect of PGA usage on the development of PCME in the perioperative period of uncomplicated cataract surgery. We hypothesized that using PGA perioperatively does not increase the incidence of PCME in patients with primary open-angle glaucoma (POAG) or OH who underwent uncomplicated cataract surgery.

Methodology

Literature Research

A systematic review was carried out, following the Preferred Reporting Items for Systematic Reviews and Meta-Analyses (PRISMA) [22]. The complete literature search was performed by two independent authors in the following electronic databases: (1) MEDLINE Ovid (1946 to March 2023), (2) Embase Ovid (1947 to March 2023), (3) Global Health (1910 to March 2023), (4) Latin American and Caribbean Health Sciences Literature Database (LILACS; 1982 to March 2023), (5) Web of Science (1975 to March 2023), (6) Cochrane Central Register of Controlled Trials (CENTRAL; 2023, Issue 2), and the (7) US National Institutes of Health Ongoing Trials Register ClinicalTrials.gov (searched in March 2023). We used a combination of keywords for prostaglandin analogs (“prostaglandin analogs,” “latanoprost,” “bimatoprost,” “travoprost,” “tafluprost”), for cataract extraction (“cataract surgery,” “phacoemulsification”), and for macular edema (“pseudophakic cystoid macular edema,” “cystoid macular edema,” and “Irvine Gass Syndrome”). A sample of the search strategy used is available in Appendix 1. The abstracts yielded in the search were screened independently by two authors (A.S. and L.V.) for eligibility according to the inclusion and exclusion criteria. The two authors cross-checked the list of the remaining articles to include any relevant articles and eliminate duplicates. Mendeley Reference Manager 2.93.0 was used to keep the references organized and identify duplicated articles. A manual check was performed by A.S. to ensure no article was eliminated purposely. The full articles were then evaluated for eligibility by the two authors, and any disagreement was resolved by discussion. The main outcome measure included the development of PCME diagnosed by clinical examination, OCT, or FA in patients with POAG or OH using PGA in the perioperative period and undergoing routine cataract surgery.

Inclusion Criteria

We included randomized controlled trials of patients aged >16 years with POAG or OH undergoing uncomplicated cataract surgery that evaluated the perioperative use of PGA and the development of PCME diagnosed by clinical examination, OCT, or FA. Patients that underwent extracapsular cataract extraction (ECCE) and phacoemulsification were included in the study, and patients with history of any glaucoma surgery were not excluded. There was no restriction regarding the type of PGA used, the language of the study, or the publication date. The perioperative period comprehends any length of time before and/or after the cataract surgery without any minimum or maximum limit on the duration of treatment with PGA.

Exclusion Criteria

The studies that were excluded are the ones that (1) include the use of nonsteroidal anti-inflammatory drug (NSAID) after surgery as prophylaxis of PCME, except if the data of a group of patients using PGA only was identifiable and could be extracted from the study, (2) include patients that presented complications intraoperatively such as posterior capsular rupture with or without vitreous loss, (3) present an association between CME and PGA use in pseudophakic patients but without any data of PGA use before or after cataract surgery, and (4) additional procedure performed that was not cataract surgery in a group of patients in the study, except if the data of a group of patients using PGA that had cataract surgery only was identifiable and could be extracted.

Data Extraction

All data were extracted and duplicated for the two authors. A standard data extraction form was used that included the study design, study size, eligibility criteria, type of participants, type of PGA used, type of glaucoma, number of eyes/patients evaluated, duration of treatment with PGA, follow-up period, and the main outcome with the diagnostic method of PCME used.

Assessment of Risk of Bias (RoB)

We used the RoB 2.0 tool [23] to assess the RoB for each of the included studies.

Results

**Figure 1:**
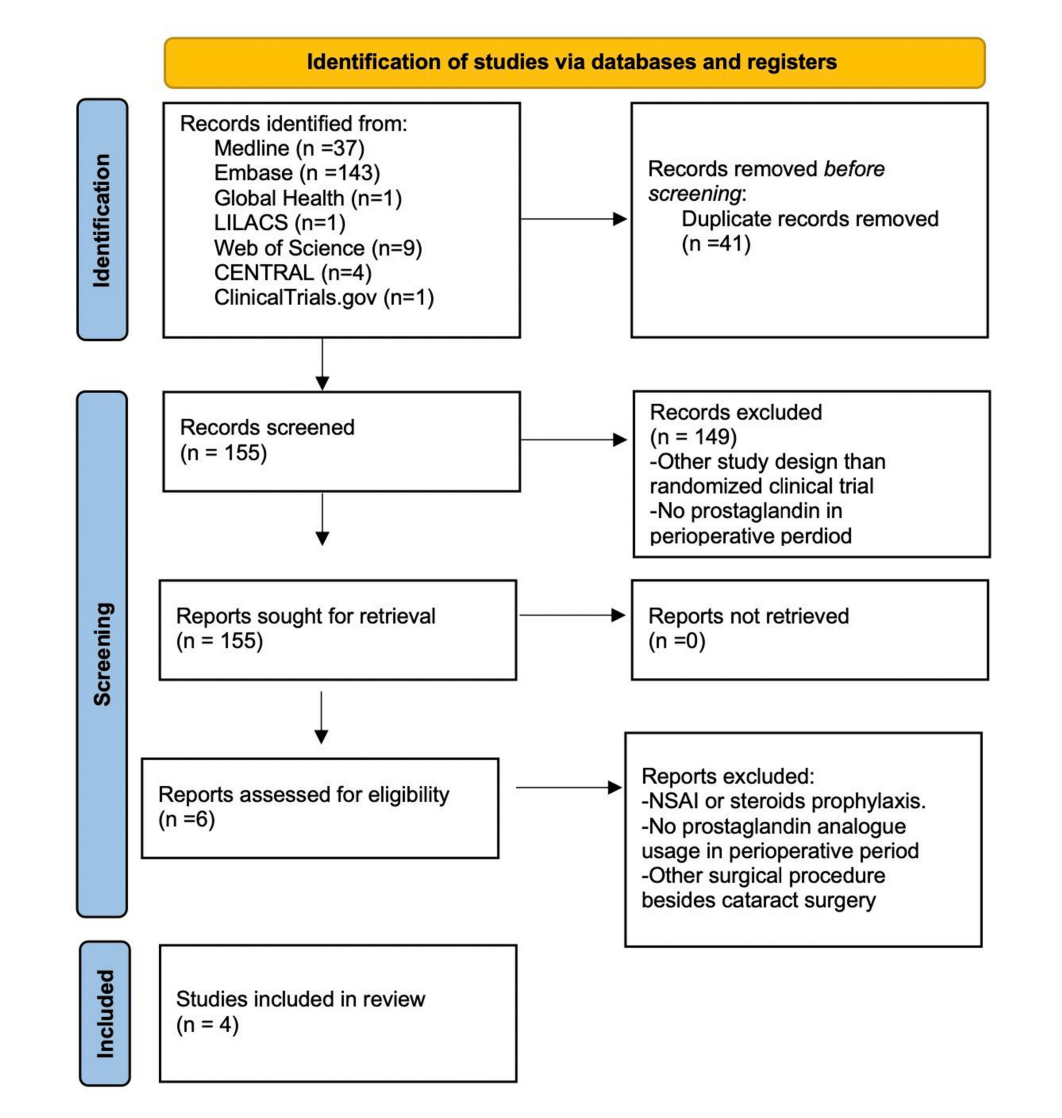
PRISMA flow diagram for randomized controlled trials

Our systematic literature search returned 196 articles. After removing duplicates, 155 unique citations were identified. We proceeded to screen the abstracts of the remaining articles, and according to the inclusion and exclusion criteria, four studies were selected for our review. A PRISMA flow diagram outlining this process is shown in Figure 1.

A summary of the included studies is given in Table 1.

**Table 1:** Description of included studies OCT: Optic coherence tomography; FA: fluorescein angiography; CMT: central macular thickness; OR: odds ratio

Study	Type of study	Participants	Intervention	Follow-up	Diagnositc method	Outcome measure	Results
Fakhraie et al. [16]	Single-masked randomized clinical trial	156 eyes (76 latanoprost group, 80 discontinued group)	Latanoprost continuation or discontinuation after uneventful cataract surgery	1 and 3 month postop	OCT	Change in CMT (increase of >50 µm)	Latanoprost group: mean CMT at baseline: 264 ± 12, 1 month postoperative: 276 ±48 (p = .03) and 3 month postoperative 271 ±55 (p = .27)
Niyadurupola et al. [24]	Single-masked randomized clinical trial	62 eyes (31 prostaglandin group, 31 discontinued group)	Prostaglandin analog continuation or discontinuation after uneventful cataract surgery	1 month postop	OCT	Presence of PCME diagnosed with cystic changes in macula with thickening of CMT from baseline	4 cases of PCME in prostaglandin group and 4 cases in discontinued group (OR = 1.0)
Arcieri et al. [25]	Single-masked randomized clinical trial	20 eyes (5 latanoprost without capsulotomy (group A), 5 latanoprost with capsulotomy (group B), 5 placebo without capsulotomy (group C), 5 placebo with capsulotomy (group D))	Latanoprost or placebo initiation after uneventful cataract surgery	1 month after treatment initiation	OCT	Distribution of normal of <5% of CMT using OCT equipment normative database.	1 case of PCME in latanoprost group without capsulotomy and none in placebo without capsulotomy (P = 1.0)
Miyake et al. [17]	Single-masked randomized clinical trial	160 eyes (latanoprost and diclofenac (group A), latanoprost and fluorometholone (group B), latanoprost placebo and diclofenac (group C) and latanoprost placebo and fluorometholone (group D)	Latanoprost or placebo initiation 2 days before surgery	5 weeks postoperative	FA	0 = no sign of fluoroscein leakage; Io= slight fluoroscein leakage; II = complete circular accumulation of fluoroscein <2 mm; III = complete circular accumulation of fluoroscein >2 mm	Incidence of angiographic PCME significantly higher in latanoprost group and fluorometholone (group B) than placebo and fluorometholone (group D) (p < .01)

Regarding the assessment of RoB, a summary of these assessments is provided in Table 2.

**Table 2:** Assessment of risk of bias by RoB 2.0 tool RoB: Risk of bias

Study	Bias arising from the randomization process	Bias due to deviations from intended interventions	Bias due to missing outcome data	Bias in measurement of the outcome	Bias in selection of the reported result	Overall risk of bias
Fakhraie [16]	Low	Some concerns	Low	Low	Low	Low
Niyadurupola [24]	Low	Some concerns	Low	Low	Low	Low
Arcieri [25]	Some concerns	Some concerns	Low	Low	Low	Some concerns
Miyake [17]	Some concerns	Some concerns	Low	Low	Low	High

Two of the randomized included clinical trials had similar methodologies. Fakhraie [16] evaluated the effect of perioperative latanoprost administration on CMT after uneventful cataract surgery in patients with glaucoma or OH. The patients were using latanoprost 0.005% eyedrops for at least six months before surgery, and patients with known risk factors for PCME were excluded, except patients with diabetes but without retinal signs. Patients with intraoperative complications (iris damage, zonular dehiscence, capsular rupture, or prolonged surgical time) were excluded, but participants who required iris manipulation with devices such as iris retractors or Y-hook were not. They also excluded patients with a topical or oral history of anti-inflammatory drug therapy two weeks before surgery. The patients were randomized into two groups: continued latanoprost and discontinued use beginning from day one postoperatively. The main outcome measure was a change in CMT between the preoperative assessment as a baseline and the one-month and three-month measurements in each group. The value to consider a significant increase was 50 μ. Of 166 eyes recruited, 156 completed the study. Five patients presented intraoperative complications, and the other five did not comply with the follow-up visits. Of the 156 patients who completed the study, 76 were in the latanoprost group, and 80 patients were in the discontinuation group.

The increase in the CMT in the latanoprost group from baseline to one month was statistically significant (p = .03), but at the three-month evaluation, it was not (p = .27). Compared to the discontinuation group, the change in CMT in the latanoprost group was not statistically significant at the one- or three-month assessment (p = .68 and p = .19, respectively). Of the secondary outcomes evaluated, clinical PCME diagnosed with fundoscopy was present in five patients (6.6%) in the latanoprost group and three patients (3.8%) in the discontinued group at one month (p = .48). At the three-month mark, 0% cases were reported in the latanoprost group and one in the discontinued group (p = 1.0). Another secondary outcome evaluated was the reduction of the IOP between both groups, which was not statistically different at one or three months (p = .08 and p = .07, respectively). Still, the decrease in the number of medications for lowering IOP was statistically significantly greater in the continued latanoprost group measured at one and three months postoperatively (p = .001 and p = .005, respectively). Finally, they measured the improvement in best corrected visual acuity (BCVA) from baseline to one and three months postoperatively, which was statistically significantly greater in the discontinuation group compared to the latanoprost continuation group at the two time points evaluated in the study (p = .01 and p = .03, respectively).

Ninyadurupola et al. similarly included patients with POAG and OH who used PGA for at least two months before cataract surgery. They included patients using any PGA, including latanoprost, tafluprost, bimatoprost, and travoprost. Patients with known risk factors for PCME and intraoperative complications were excluded. As opposed to Fakhraie et al., patients requiring any iris manipulation maneuvers intraoperatively and diabetic patients with or without retinal signs were also excluded. Sixty-two eyes were recruited and randomized into two groups: continued use of PGA and the discontinued PGA group, beginning the day of the surgery. All patients had a macular OCT preoperatively at baseline and one-week and one-month appointments. The main outcome measure was the presence of PCME on OCT at one-month postoperative evaluation, diagnosed by cystic changes in the macula with thickening of CMT from baseline. In this study, no cases of PCME were detected at the one-week assessment in either group. At one-month follow-up, PCME was detected in four eyes (12.9%) in the continued PGA group and four eyes (12.9%) in the discontinued PGA group (OR: 1.0, 95%CI: 0.23-4.41). All four types of PGA were associated with PCME, not presenting more commonly in one kind specifically. Of the patients that developed PCME, the increase in CMT from baseline between the two groups was not significantly different (p = 0.21), and all increases of CMT in eyes that developed PCME were less than 52 μ, except for one eye that presented an increase of 260 μ. The measurements of the IOP between the two groups at one week were similar to the baseline, and there was no significant difference between the IOP measurements of the two groups. Still, at one month, there was a significant reduction in IOP compared with baseline in the continued prostaglandin group (p = 0.003) and not in the discontinued group. They reported that the unaided visual acuity in the patients that developed PCME was similar in the two groups at one month. Still, they did not report visual acuity improvement from baseline to the one-month postoperative measurement. Finally, the duration of PGA treatment previously from surgery (p = 0.594), number of glaucoma treatment medications (p = 0.299), therapy with drops containing preservatives such as BAK (p = 0.111), and phacoemulsification time needed in the surgery (p = 0.490) were not associated with the presence of PCME at one month postoperatively.

Arcieri et al. [25] evaluated patients with a history of cataract surgery in the last six months without known risk factors for developing PCME. Unlike the two studies described previously, the patients were not previously exposed to PGA and started therapy with PGA or a placebo when the study began. The patients were randomized into four groups: latanoprost without capsulotomy (group A), latanoprost with capsulotomy (group B), placebo without capsulotomy (group C), and placebo with capsulotomy (group D). Twenty patients were included in the study, with five allocated to each group. The patients in groups B and D were excluded from our analysis since another procedure was performed other than cataract surgery, and the patients in groups A and C were included since these patients only had cataract surgery, and their data could be identified and extracted from the report. All patients were assessed before initiating treatment, at 15 days, and at one month after treatment initiation. At all visits, macular OCT was performed. The main outcome measure was the presence of PCME diagnosed with a distribution of normal macular thickness <5% using the normative equipment database. The results reported one case of PCME in the latanoprost group without capsulotomy (group A) and zero in the placebo without capsulotomy (group C) (p = 1.0). None of the patients reported diminished visual acuity or alterations such as metamorphopsia, making the diagnosis of subclinical PCME in these patients. Of the secondary outcomes measured, the CMT in the latanoprost and placebo groups was not significantly different at the 15-day evaluation (p = 0.14). Still, at the 30-day assessment, the latanoprost group had a significantly higher measure of the CMT (236.4 ± 29.4 μm in the latanoprost group and 197.8 ± 19.3 μm in the placebo group (p = 0.039)).

Finally, the study by Miyake et al. [17] evaluated the incidence of angiographic PCME in eyes receiving latanoprost 0.005% or placebo. They also aimed to assess the effect of diclofenac as prophylaxis for PCME. They recruited 160 eyes in total and allocated 40 eyes to four groups: latanoprost and diclofenac (group A), latanoprost and fluorometholone (group B), placebo and diclofenac (group C), and placebo and fluorometholone (group D). The patients in groups A and C were excluded from our analysis since an NSAID was used as prophylaxis for PCME, and patients in groups B and D were included since these did not receive NSAID after cataract surgery, and their data was identifiable and extracted from the report. Due to follow-up requirements, 37 patients in groups B and D each were included in the study at the end. To note, the exclusion criteria in this study did not include patients with known preoperative risk factors for PCME, and they didn't need to have a normal macula fundoscopy anatomy to be included in the study. Treatment with latanoprost or placebo was started two days before surgery and continued in the five-week follow-up period of the study. An FA was performed in the fifth week postoperatively to detect the presence of PCME. The grading system to evaluate the outcome measure of the presence of PCME was the following: 0 = no sign of fluorescein leakage; I = slight fluorescein leakage into the cystic space; II = complete circular accumulation of fluorescein in the cystic space, but it measures less than 2 mm; and III = entire circular accumulation of fluorescein larger than 2 mm. The incidence of angiographic PCME was significantly higher in the latanoprost and fluorometholone (group B) than in the placebo and fluorometholone (group D) (p < .01). For the secondary outcomes, they measured the BCVA at one day, three days, one week, two weeks, and five weeks postoperatively and found no significant difference on the measurements between the groups after surgery. They also included the mean ± SD IOP reduction measurement and found that for both groups, there was a significant reduction of the IOP from baseline at the five-week postoperative evaluation, but most importantly, this reduction was significantly higher in the latanoprost group than the placebo (15.8 ± 3.3 in the latanoprost group and 18.2 ± 3.5 in the placebo group, p = <.05)

Discussion

The occurrence of PCME is one of the leading causes of visual loss in the early postoperative period of cataract surgery, presenting a peak incidence in the first five weeks after the procedure [6]. Most practitioners consider PCME to be a more benign retinal condition that, with correct treatment, has a complete resolution with the restoration of visual acuity. In reality, PCME can be a condition that carries visual morbidity. In a large retrospective database study of 81, 984 eyes, patients who developed PCME had a highly significant difference regarding visual acuity at the end-point measurement at 24 weeks postoperatively [7]. Many risk factors for its occurrence have been identified, but using PGAs in the perioperative period remains controversial. 

The lens capsule acts as a barrier between the anterior and posterior segments and helps maintain the integrity of the anterior segment [26,27]. By removing the lens surgically and implanting an artificial lens, we can produce stress in other tissues of the anterior segment, causing increased production of COX2, PGE2, and other inflammatory mediators [28], adding a disruption of the blood-aqueous barrier that occurs in the ciliary muscle that causes the expected aqueous humor flare after cataract surgery. The inflammatory mediators can diffuse to the posterior segment and disrupt the blood-retinal barriers, causing fluid leakage and edema in the macula [26,29,30]. Different theories have been proposed to explain the possible association between PGA therapy and the occurrence of PCME, including that PGA causes a direct alteration of the blood-aqueous barrier that increases the secretion of inflammatory mediators. Through different studies, we can learn that PGA does not disrupt the blood-aqueous barrier directly [26] but prolongs the production of inflammatory mediators in pseudophakic eyes, which could increase their migration to the posterior segment and start an inflammatory cascade that ends with PCME. In uncomplicated cataract surgery, the temporary increase of endogenous PG with the addition of topical PGA can cause mild asymptomatic CME detected by OCT or FA [14,19]. Conversely, after complicated cataract surgery or even in an uncomplicated one, the cumulative effect of topical PGA with the temporary increase of endogenous PG can cause clinically significant PCME to become more prevalent [6,15].

Systematic reviews of observational, retrospective, and prospective cohort studies have evaluated the association of PGA use in the perioperative period of cataract surgery with the incidence of PCME. The results have been conflicting, some reporting an association [14,15,31] and others not [18,20,32], but according to the Levels of Evidence from the Centre for Evidence-Based Medicine of the University of Oxford [33], these studies are allocated in the level 2A and 2B category of evidence. A systematic review of randomized clinical trials (RCTs) would be allocated in a level 1A category of evidence, and such a study could help to elucidate this subject since confounding factors that could not be controlled in the other study designs could result in conflicting results.

The four studies that resulted from the systematic review of RCTs evaluated the incidence of PCME in patients undergoing cataract surgery and using PGAs in the perioperative period. Two of the studies had similar methodologies, apart from a few details. Fakharaie and Ninyadurupola recruited patients using PGA for at least six and two months, respectively, and allocated patients to a PGA continuation group and a PGA discontinuation group. The main outcome measure was the presence of PCME in both groups at one month and three months postoperatively, but it was measured differently. Fakharaie established a more objective measure to determine the presence of PCME, with an increase of the CMT of three standard deviations (SDs) above the mean CMT of healthy subjects, which translates into a 20% increase or >50 μ, which was presented with acceptable sensitivity and specificity to diagnose PCME by Hee et al. [34]. On the other hand, Ninyadurupola diagnosed PCME with the presence of cystic changes in the macula with thickening of CMT from baseline, but they did not present a cutoff value. Both excluded patients with known risk factors for PCME and alterations in the morphology of the macula OCT preoperatively, but Fakharaie did not exclude patients who needed iris manipulation, which is an important factor of the postoperative inflammation that could contribute to PCME. They also did not exclude patients with diabetes but without retinal signs to present a more “clinically interesting study” [16] that could be more easily applied to our usual clinical setting. This could represent a confounding factor since it has been established that diabetes is a known risk factor for developing PCME, even in patients without retinal signs of diabetic retinopathy [7]. Both RCTs did not find a measurable effect of CMT by PGA in the perioperative period in patients undergoing cataract surgery. They advised that there was no need to suspend PGA therapy in patients without known risk factors for developing PCME and with no intraoperative complications.

Arcieri and Miyake had different methodologies in their studies. Arcieri included patients not on PGA therapy before surgery and started PGA therapy or a placebo in the six-month postoperative period of the study. They defined PCME as a normal distribution of <5% in CMT utilizing a normative database from the OCT one month after treatment initiation. They did not report a difference in the incidence of PCME between the group that initiated PGA and placebo, and the one patient that developed PCME in the latanoprost group was a subclinical diagnosis since the patient did not complain of any visual disturbances and did not present with alterations in visual acuity. They also measured CMT from baseline and at one month after treatment initiation, and even if they found a significant difference between both groups, measuring more in the latanoprost group, the mean increase was not more than 50 μ, and if we apply the more objective definition of PCME used in the study by Fakharaie, the diagnosis of PCME would have not been done in these patients.

On the other hand, Miyake evaluated the incidence of PCME in patients who initiated latanoprost or placebo two days before surgery and had FA at five weeks postoperatively. They reported a significant increase in the incidence of PCME in the latanoprost group by FA. Still, most of the patients in the latanoprost group were in the grading I group, which is described as a slight leakage of fluorescein in the cystic space. It is important to mention that they did not exclude preoperatively patients with macular morphology alterations, which could indicate that some of the patients had macular disease before the surgery and initiation of latanoprost. Also, FA was the study of choice to evaluate PCME in the past. This explains why it was the diagnostic method used in the study by Miyake et al. in 1999. Nowadays, we know that the evaluation made by FA is purely qualitative, and OCT has a much higher sensitivity than FA to detect PCME with the possibility to perform quantitively evaluations of the CMT.

An important factor that differed between the studies was the postoperative protocol of corticosteroids which could also influence postoperative inflammation. Fakharaie chose an intensive protocol of betamethasone 1.0% every two hours for one week, which was tapered afterward, Miyake chose a much less intensive one of topical fluorometholone 0.1% 4x at the day of surgery, then 3x per day for four weeks. Ninyadurupola applied a scheme closer to the everyday practice of topical dexamethasone 0.1% 3x daily for three weeks. The postoperative control of intraocular inflammation could impact the incidence of PCME. More postoperative flare translates into a greater alteration of the blood-aqueous barrier. This could mean more migration of inflammatory mediators to the posterior segment and alteration of the blood-retinal barrier that could induce PCME. Controlling the postoperative inflammation response with corticosteroids and NSAID is known to diminish the incidence of PCME [35].

An important matter regarding discontinuing PGA in the perioperative period is its impact on the IOP control in these patients since the major reduction of IOP by medication is accomplished by PGA [35]. In the study by Fakharaie et al., there was a greater reduction of IOP in the latanoprost group, but it was not statistically different. On the other hand, the reduction in the number of medications was statistically significantly greater in the latanoprost group. Ninyadurupola reported a significant reduction in IOP at one month in the PGA continuation group compared to baseline, and Miyake encountered a significant reduction in IOP at five weeks postoperatively from baseline in the latanoprost group with a statistically significant reduction of IOP compared to the placebo group. These findings go in hand with what is reported in the literature regarding the effectiveness of PGA treatment, and the reduction of IOP could mean a difference in the progression of glaucoma in some patients, especially those with advanced disease. This could imply a clinical repercussion of suspending PGA in patients with difficult-to-control IOP.

The visual acuity (VA) measurements in the different groups were a secondary measure. In the study by Fakhraie et al., the improvement in BCVA was lesser in the latanoprost group. The difference was greater at one month than three months postoperatively, meaning that the normalization of VA could be expected with time and correct treatment of the PCME. Still, an important confounding factor that was not considered was the nuclear density of cataracts that could impact the improvement of BCVA postoperatively.

There were limitations in our systematic reviews inherent in the different methodologies of the RCTs. We can assess qualitatively that there is a difference in the definition of PCME and the diagnostic method utilized, the reason why we did not performed a meta-analysis. The variability in the definition of the outcome measure and the diagnostic method utilized is expected to lead to differences in the observed intervention effects, producing an important statistical heterogeneity. A meta-analysis should only be performed when the group of studies in a systematic review is homogenous in participants, interventions, and most importantly outcome measures. If there is considerable variation and inconsistency in the direction of the effect, it may be misleading and erroneous to present an average value for the intervention effect. Also, we had to analyze some studies based on the extracted data and disregard groups within the study because it included NSAID prophylaxis or a procedure other than cataract surgery performed; this could impact the power of the study since it was calculated based on the inclusion of this group of patients. On the other hand, the studies had different follow-up periods, the most common being one-month postoperatively. However, as reported in the literature, the peak incidence of PCME is at five weeks postoperatively, so a longer follow-up period could have lightened different results. Future prospective clinical trials with homogenous main outcome measures using the same diagnostic method and criteria for PCME could be under the findings of this systematic review.

## Conclusions

In conclusion, the results of the present systematic review of randomized controlled clinical trials show no causal relationship between PCME in patients using PGA undergoing uneventful cataract surgery and suggest that they do not have to be suspended in patients without known risk factors of PCME or intraoperative complications. It would also be sensible to suspend PGA in this group of patients to diminish the number of exogenous PGA administered that could start or add to a major inflammatory cascade in these patients.

## Appendices

Appendix 1

Search history for MEDLINE database using Ovid platform

MEDLINE

Ovid MEDLINE(R) <1946 to March week 4 2023>

1          Macular Edema/       8796

2          macular edema.mp. 12483

3          pseudophakic cystoid macular edema.mp.         178

4          cystoid macular edema.mp.           2868

5          1 or 2 or 3 or 4          12483

6          Prostaglandins, Synthetic/  1202

7          prostaglandin analogs.mp. 416

8          Latanoprost/  1571

9          latanoprost.mp.        1920

10        Bimatoprost/ 586

11        bimatoprost.mp.       766

12        Travoprost/    483

13        travoprost.mp.          643

14        6 or 7 or 8 or 9 or 10 or 11 or 12 or 13     3760

15        cataract surgery.mp.           19328

16        Cataract Extraction/ 26077

17        cataract extraction.mp.       28141

18        Phacoemulsification/           11637

19        phacoemulsification.mp.     13966

20        extracapsular cataract extraction.mp.      1929

21        15 or 16 or 17 or 18 or 19 or 20    42709

22        5 and 14 and 21       37
